# Ultrastructural changes in oocytes during folliculogenesis in domestic mammals

**DOI:** 10.1186/s13048-014-0102-6

**Published:** 2014-10-30

**Authors:** Fernanda Paulini, Renata Carvalho Silva, José Luiz Jivago de Paula Rôlo, Carolina Madeira Lucci

**Affiliations:** Physiological Sciences Department, Institute of Biological Sciences, University of Brasília, Brasília, DF Brazil

**Keywords:** Preantral follicle, Cortical granules, Zona pellucida, Lipid droplets

## Abstract

The ultrastructural analysis of oocytes and ovarian follicles has been used to evaluate the effects of assisted reproductive techniques, such as cryopreservation or *in vitro* oocyte maturation. It also benefits the understanding of such complex mechanisms that occur during folliculogenesis. From the beginning of primordial follicles growth until oocyte maturation in preovulatory follicles oocyte cytoplasmic organelles undergo dynamic alterations that reflect physiological changes and development. This review aims to make a retrospective survey of the relevant features of follicles and oocytes ultrastructure, highlighting the differences between mammalian species, specially the domestic ones.

**Electronic supplementary material:**

The online version of this article (doi:10.1186/s13048-014-0102-6) contains supplementary material, which is available to authorized users.

## Introduction

Female mammals have hundreds of thousands of oocytes already at the time of birth. The ovarian cortex contains follicles at different developmental stages [1],[2]; these can be classified according to size, type and number of granulosa cells, or if they are dependent or not on gonadotrophic hormones. The follicles are named preantral or antral follicles, according to the absence or presence of a cavity, respectively. Preantral follicles are usually classified in three stages: primordial, primary or secondary follicles [3]. At the antral stage, most follicles undergo atretic degeneration [4]. However, a few of them reach the preovulatory stage under gonadotropin stimulation. The fate of each follicle is controlled by endocrine and paracrine factors [5],[6]. The complete development of the follicle culminates in ovulation, which is when the mature cumulus-oocyte complex is released and may be fertilized. Although many studies have focused on the hormonal regulation of the development of large antral follicles, few studies have focused on follicle development at the early stages [7]-[9].

As follicles and oocytes develop, many changes in their ultrastructure and physiology occur. In fact, there are many papers describing these morphologic changes. This knowledge is important to understand the physiology of female germ cells. This review describes the morphological changes that occur during oocyte and follicular growth and differentiation in different mammalian species, with special focus on domestic species.

### Origin and establishment of ovarian follicles

Germ cells that originate the pool of primordial oocytes derive from the inner cell mass of the developing blastocyst [10]. They arise in the allantois and migrate into the endoderm and to the genital ridge [11]. During their migration the germ cells divide mitotically and increase in number [12]. Proliferation of the coelomic epithelium and concomitant condensation of the underlying mesenchyme lead to the formation of a swelling, denominated genital ridge or gonadal crest [13]. Initially, the gonadal crest does not contain any primordial germ cells, which at that time are still located in the epithelium of the yolk sac, close to the base of the allantois. A migratory phenotype of the primordial germ cells reaches the gonadal crests through amoeboid movements [13]. Once established in the developing ovary, the proliferating primordial germ cells begin to differentiate into oogonia [12]. The population of oogonia expands through a predetermined species-specific number of mitotic divisions until the cells enter meiosis and become oocytes [14],[15]. The maximum number of female germ cells is reached at the time of transition from mitosis to meiosis [16]. The maximum number of germ cells in some species can be seen in Table 1. Although Johnson et al. [17] demonstrated that primordial germ cells are present on the surface epithelium of the ovary, there is still controversy about whether the reserve of oocytes is renewable or not [18]. Recently, White et al. [19] isolated the so-called "rare mitotically active germ cells" from adult mouse and human ovaries and propagated them *in vitro*, which after all generated oocytes.

**Table 1:** **Maximum number of female germ cells reached in fetal ovaries during gestation in different species and the number of germ cells in the ovaries at the time of birth or nearly after**

Species	Maximum number of germ cells(Day of gestation)	Number of germ cells close after birth(Day after birth)
Calf [20]	2,700,000 (110)	68,000 (13 days after birth)
Pig [21]	1,100,000 (50)	500,000 (at birth)
Buffalo [22]	23,540 (210)	20,000 (at birth)
Rat [23]	75,000 (18)	27,000 (2 days after birth)
Human [24]	6,800,000 (150)	2,000,000 (at birth)

The first oogonia to undergo meiotic division are located in the innermost areas of the ovarian cortex and the developmental wave of meiosis spreads outwards. By mid- to late-gestation in large animals and humans many stages of germ cell development are simultaneously present in the fetus' ovary [10]. Clusters of germ cells are formed with a number of oogonia and surrounded by somatic cells that are considered granulosa cell precursors [12],[25].

Folliculogenesis concerns to a lengthy developmental process a follicle goes through, from the time it leaves the reserve pool and begins to grow by cell proliferation and antrum formation until ovulation or atresia [26],[27]. Folliculogenesis starts before birth in some mammalian species (cow, sheep and buffalo) [28] or shortly after birth in others (mouse, rat, hamster) [28]-[30]. By this time all germ cells in the ovaries are primary oocytes, which will remain in this stage until puberty, when at each cycle selected follicle(s) go on to ovulate [10].

Even before birth, some oocytes will die by a process named apoptosis. Apoptosis is likely to be a mechanism for reducing the number of oocytes/ovarian follicles, and females are born with far fewer oocytes than the maximum number reached during fetal life [31] (Table 1).

The supply of preantral follicles per ovary is highly variable among species [20] and has been estimated at 70,576 in *Bos indicus*[32] and 89,577 in *Bos Taurus*[33], 19,819 in buffaloes [34], 75,642 in sheep [35], 37,646 in goats [36], 402,000 in humans [37], 106,071 in monkeys (*Cebus apella*) [38], 37,853 in domestic cats [39], 210,00 in pigs [40] and 47,900 in domestic dogs [41].

Every day, a great number of primordial follicles initiate growth, granulosa cells proliferate and oocytes start developing [42]. The initiation of primordial follicles growth starts a series of morphological changes leading to subsequent stages of follicular development - the primary and secondary follicles (preantral), tertiary and, finally, the preovulatory follicles (antral) [43]. These changes can be observed in follicular and oocyte diameter and the number of granulosa cells (Table 2). Alterations in follicular and oocyte ultrastructure and physiology will happen at many levels, and there are some distinct modifications among mammalian species.

**Table 2:** **Differences among species in follicle diameter**, **oocyte diameter and number of granulosa cells**

Species	Follicular diameter (μm)			Oocyte diameter (μm)			Mean number of granulosa cells		
	PL	PR	S	PL	PR	S	PL	PR	S
Cattle [44]	36	49	88	28	32	44	7	15	62
Buffalo [45]	35	42	53	25	27	29	4-8	8-20	-
Sheep [46]	41	75	129	35	52	73	16	128	637
Goat [47]	20	24	44	16	17	25	6	11	31
Cat [39]	28	41	75	23	30	41	7	13	46
Dog [41]	28	43	102	22	28	48	6	15	62
Human [48]	35	42	77	32	32	48	13	52	360
Pig [49]	34	40	85	26	27	39	5	8	50

*PL*: primordial follicle, *PR*: primary follicle, *S*: secondary follicle.

### Structure of primordial follicle and initiation of growth

Primordial follicles are characterized by a quiescent oocyte, arrested in prophase I of meiosis surrounded by a single layer of flattened granulosa cells. These primordial follicles constitute the ovarian reserve from which follicles are engaged for development [50].

**Figure 1:**
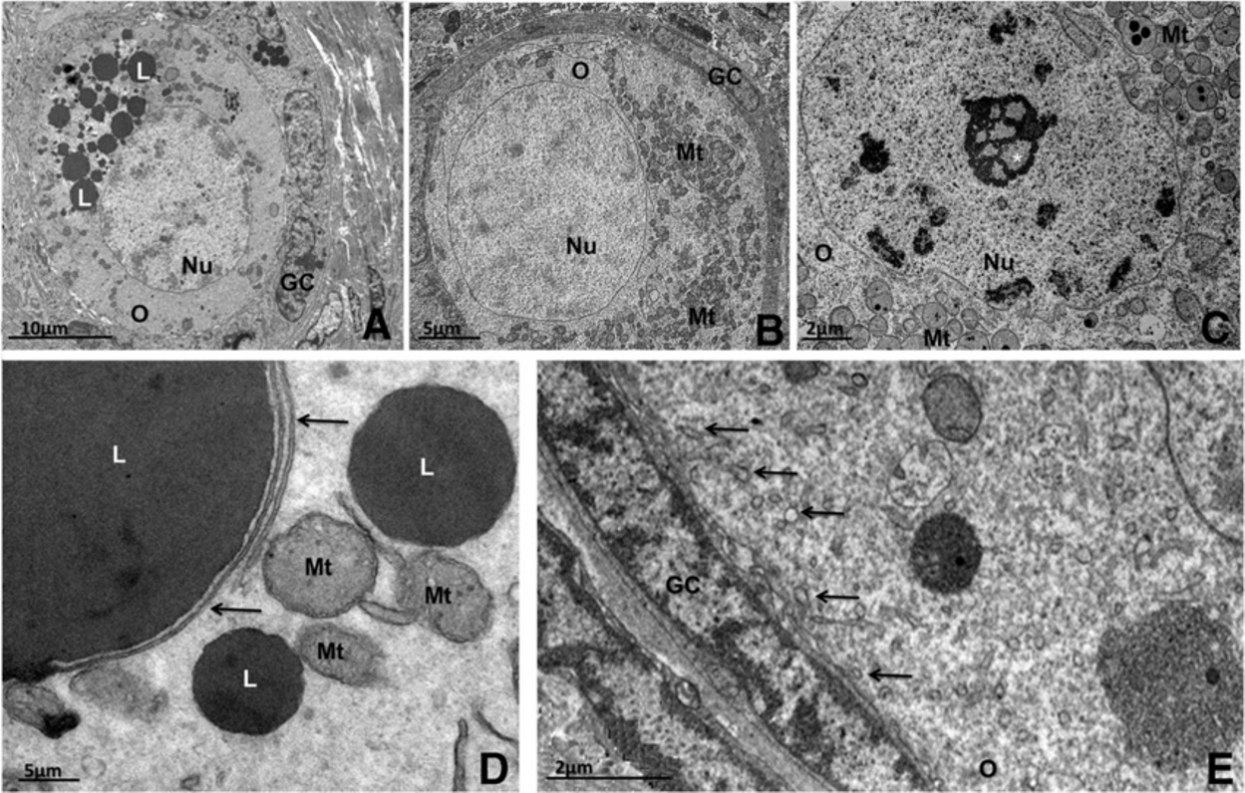
**Transmission electron micrographs of primordial follicles. A**: Pig primordial follicle with central nucleus and a large amount of lipid droplets at one pole of the oocyte. **B**: Bitch primordial follicle with peripheral nucleus. Note the abundance of round mitochondria homogeneously spread throughout the ooplasm. **C**: A representative nucleolus in the oocyte nucleus from a cattle primordial follicle. **D**: Detail of the association among lipid droplets, smooth endoplasmic reticulum (arrows) and mitochondria in pig oocyte. **E**: Detail of the close contact between granulosa cell and oocyte in cat primordial follicle showing many coated pits (thin arrows) in the cortical ooplasm. O: oocyte, Nu: nucleus, GC: granulosa cells, L: lipid droplet, Mt: mitochondria, *: nucleolus.

The quiescent oocytes are ovoid or spherical with a homogeneous cytoplasm. The nucleus may be located in a central or eccentric position inside the oocytes in most species (Figure 1A and B). The nucleus is enclosed by a smooth envelope [51],[52]. Usually, the chromatin is found uncondensed and one or two nucleoli are observed (Figure 1C) [44],[49],[52],[53].

In most species, the cytoplasm of oocytes in primordial follicles exhibits organelles close to the nucleus or uniformly distributed throughout the cytoplasm (Figure 1A and 1B). In humans, groups of organelles are seen close to the nucleus and are named Balbiani bodies [54]. Balbiani body is a large distinctive collection of organelles asymmetrically located near the nucleus in very young oocytes, consisting of mitochondria and associated endoplasmic reticulum surrounding Golgi elements. Besides being well described in human oocytes, they are also found in oocytes of other species (vertebrates and invertebrates). Although the function of mammalian Balbiani body is unknown, this structure may have a possible role in nucleo-cytoplasmic transfer [55],[56].

In any case, the most abundant organelles found in primordial follicle oocytes are round-shaped mitochondria (Figure 1B) [44], which are known to be an immature form of this organelle and develop to an elongated shape as they become mature [57]. The presence of immature mitochondria is consistent with primordial follicles containing a quiescent oocyte that does not require a large amount of energy to survive [44]. An abundant, scattered mitochondrial population is evident in primordial follicle oocytes in pigs and numerous mitochondria are randomly distributed, with an extensive network of endoplasmic reticulum permeating the cytoplasm [58]. In cows primordial follicle oocytes, round mitochondria are abundant and they present few peripheral cristae [44]. In yaks, a few hooded mitochondria are observed [52].

Besides mitochondria, in most mammals the ooplasm of the primordial follicle contains lipid droplets, endoplasmic reticulum, some Golgi cisternae, polyribosomes and a variable number of vesicles [57]. In non-domestic cats, the endoplasmic reticulum is not well developed and Golgi complexes are rarely seen [59]. In the ooplasm of buffaloes, a delimited region with a well-developed smooth endoplasmic reticulum is observed [45]. In yaks [52] and pigs [49], polyribosomes are seen on the surface of the rough endoplasmic reticulum and distributed throughout the ooplasm.

The oocytes of all mammals contain lipids, and the content varies between species in terms of abundance and characteristics. Especially in pigs, lipid droplets are abundant in the oocytes from the primordial stage onwards, and they appear as small dark round structures (Figure 1A) [49]. Lipid droplets are considered to be an energy source [60]. In most species, often the endoplasmic reticulum, mitochondria and lipid droplets are found associated with each other (Figure 1D) [57]. Some early biochemical studies showed that the synthesis of lipids (such as the triacylglycerol stored on lipid droplets) requires enzymatic activity associated with both the endoplasmic reticulum and mitochondria, with lipids being transported and transferred between the endoplasmic reticulum and mitochondria (For a review see [61]). As the follicle grows, the number of these metabolic units in the ooplasm increases, denoting a rise in oocyte metabolism [34]. In goats, buffaloes and sheep, many vesicles are spread throughout the cytoplasm and they present different electron densities [45],[47],[62], which might mean different contents, like proteins or mucopolysaccharide [63].

In primordial follicles, granulosa cells are small and have a relatively large nucleus that matches the cell format, and presents clusters of condensed and uncondensed chromatin [44]. In goats, granulosa cells present low density of cytoplasmic organelles [47], and in buffaloes scarce myelin figures are present [45], being the result of the digestion of old or nonfunctional structures [64].

Overall, there are no specialized junctions between granulosa cells or between them and the oocyte. At this stage, any substance that needs to gain access to the oocyte is incorporated by endocytosis or enters by diffusion through intimate contact between the membranes of granulosa cells and the oocyte. This can be observed by the presence of a large number of coated pits in the cortical cytoplasm of primordial follicle oocytes of bovine (Figure 1E) [44],[57],[65],[66] and other species [47],[52].

Initiation of growth and the transition from primordial to primary follicle begins with the development of primordial follicles. At this point, follicles become "committed", and follicular growth proceeds until the follicle is ovulated or undergoes atresia [50],[67]. Follicular growth takes place in only a small number of follicles each time [68], and the complete elucidation of the factors responsible for triggering follicular development remains one of the major unsolved problems of ovarian physiology.

**Figure 2:**
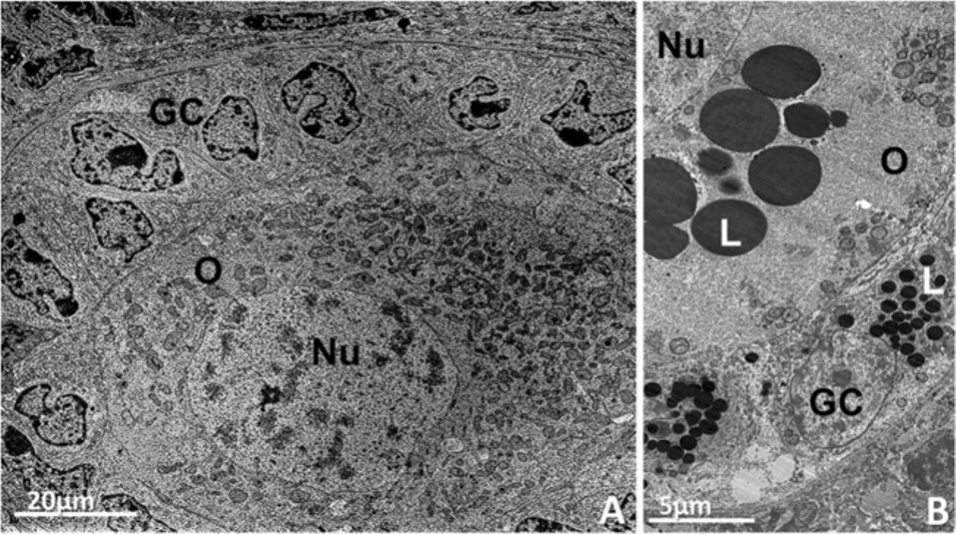
**Primary follicles. A**: Bovine primary follicle showing the oocyte with organelles homogeneally distributed throughout the cytoplasm surrounded by cuboidal granulosa cells. Round and elongated mitochondria can be observed. **B**: Pig primary follicle with several lipid droplets in the oocyte and granulosa cells cytoplasm. O: oocyte, Nu: nucleus, GC: granulosa cells, L: lipid droplet.

The classical changes that characterize this process are the differentiation and proliferation of granulosa cells and the enlargement of the oocyte: in the primary follicle, granulosa cells increase in number and become cuboidal in shape [2]. Granulosa cells at this stage are situated close to each other and adherens junctions are common between granulosa cells and the oocyte and also between adjacent granulosa cells [57]. Their nuclei are irregular with indentations and there are round mitochondria, endoplasmic reticulum, few Golgi cisternae and vesicles in their cytoplasm [2],[47] (Figure 2A). Additionally, in pig primordial follicles many lipid droplets can be seen in the oocyte and granulosa cells (Figure 2B). The oocyte undergoes volume expansion, the zona pellucida proteins start to be secreted between the growing oocyte and the granulosa cells in cattle [2] and buffaloes [45], and an evident zona pellucida is observed at the primary follicle stage in some species, including rats [69], mice [11],[70], guinea pigs [71], rabbits [72], rhesus monkeys [73], humans [74],[75], sheep [62], domestic cats [39], and non-domestic cats [59].

In general, most ultrastructural features of the ooplasm and its organelles and inclusions of primary follicles are similar to those described for the primordial follicles. Most mitochondria are still round, although elongated and dividing mitochondria become more common [57] (Figure 2A).

### From primary to secondary follicles

Once the primary follicle starts developing this process cannot be interrupted, and many morphological changes will happen in the oocyte and granulosa cells during the further steps of folliculogenesis [76].

The organelles that were uniformly distributed throughout the ooplasm in primordial and initial primary stages migrate to the periphery of ooplasm in secondary follicles, leaving an organelle-free zone next to the nucleus [49]. In cats, the organelles are organized in clusters [39], such organization will only happen later in other species [66],[77].

Oocytes of secondary follicles are predominantly spherical and present a cytoplasm with vesicles and round and elongated mitochondria in cows [44],[57],[78], sheep [79], goats [36],[47],[80], cats [39], buffaloes [34],[45], humans [54],[81] and yaks [52].

**Figure 3:**
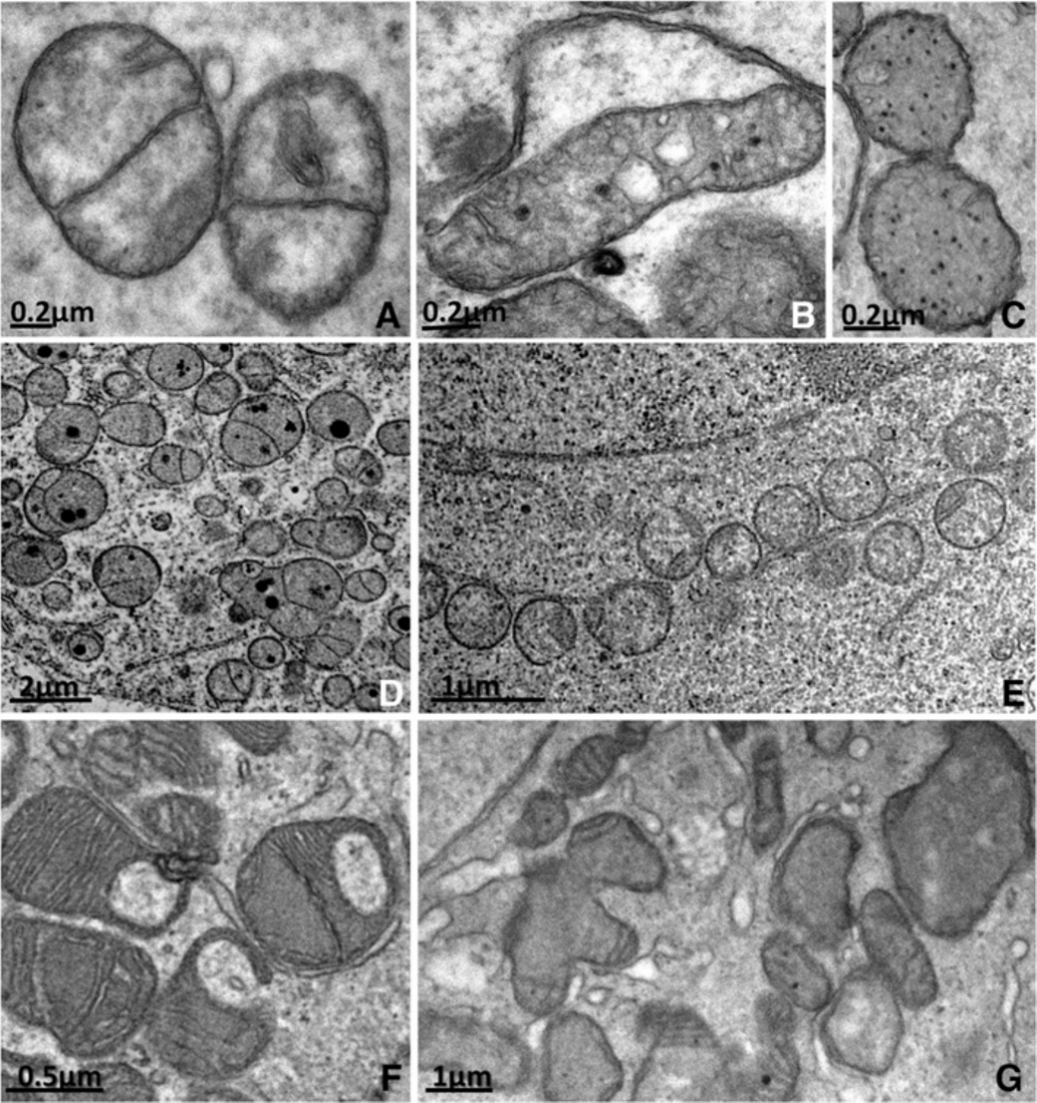
**Types of mitochondria observed in oocytes of mammalian species. A**: Round (from pig). **B**: Elongated (from pig). **C**: Round with peripheral cristae (from pig). **D**: Round mitochondria with electron-dense granules inside (from cattle). **E**: Round mitochondria arranged as "string of pearls" (from bitch). **F**: Hooded mitochondria (from cattle). **G**: Pleomorphic mitochondria with cristae arranged parallel and close to the outer membrane (from goat).

Mitochondria are still the most abundant organelle in secondary follicle oocytes. Although round mitochondria (Figure 3A) are still present, their elongated form (Figure 3B) becomes more frequent, which is consistent with the higher metabolism of the oocytes at this stage. In buffaloes and pigs, however, round mitochondria are still more abundant in secondary follicle and elongated mitochondria are rare [45],[49]. Two types of round mitochondria can be observed in oocytes from cats [39] and other species, those with low electron-density and few peripheral cristae (Figure 3C) and those with high electron-density and many cristae. In cows and buffaloes, mitochondria presenting a membrane dividing their matrix into two or more compartments are often seen [44],[45], which may denote organelle division [44]. In goat oocyte mitochondria a few cristae are arranged parallel and close to the outer mitochondria membrane, leaving a large central area of moderately electron-dense inner matrix [47]. In pigs and cows, electron-dense granules are often observed in the mitochondrial matrix (Figures 3B, 3C and 3D) [44],[49],[57],[82]. These electron-dense granules in the mitochondrial matrix are very common in some cell types and have been reported to be especially prominent in tissues transporting large amounts of ions or water, suggesting that these granules are related to the regulation of the internal ionic environment of the mitochondrion [64]. Silva et al. [49] showed that round mitochondria in pig secondary follicles were organized as "strings of pearls" (Figure 3E), which can also be observed in other species [41]. Hooded mitochondria (Figure 3F) as well as pleomorphic forms (Figure 3G) can also be seen in the secondary follicle oocytes of sheep [83],[84], cattle [85] and yaks [52].

**Figure 4:**
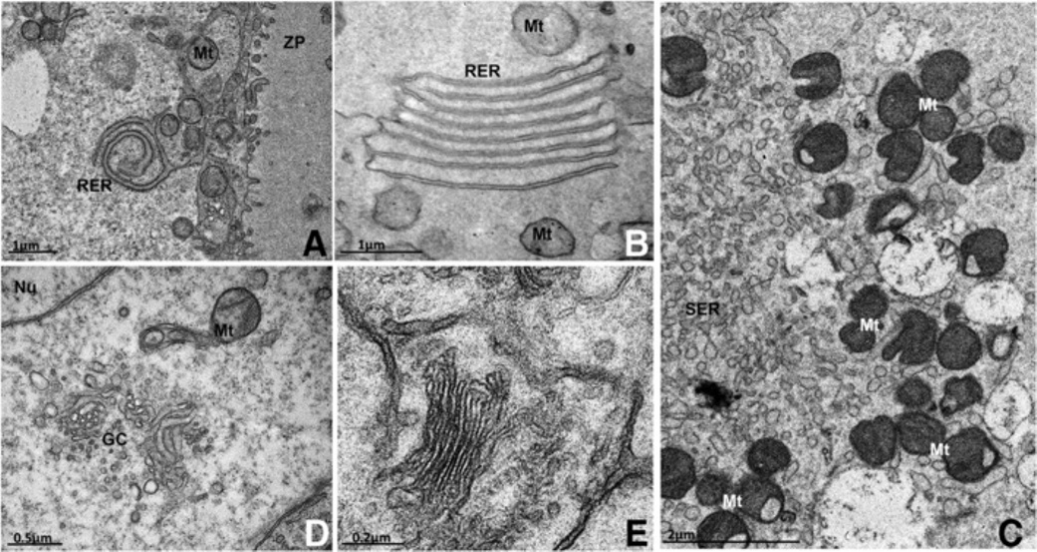
**Well**-**developed rough** **(A and B)** **and smooth** **(C)** **endoplasmic reticulum and Golgi complex** **(D and E)** **in secondary follicle oocytes.** RER: rough endoplasmic reticulum, SER: smooth endoplasmic reticulum, Mt: mitochondria, GC: Golgi complex, Nu: nucleus, ZP: zona pellucida.

Endoplasmic reticulum (Figure 4A, 4B and 4C) and Golgi cisternae (Figure 4D and 4E) become aggregated and well developed, which is also consistent with the higher metabolism of the oocytes in growing follicles. There are also a lot of free polyribosomes and a larger amount of lipid droplets [65]. Myelin figures are commonly observed in the ooplasm [44], suggesting the turnover of cytoplasmic structures [64]. In pigs, lipid droplets are abundant and they change in appearance from small round dark droplets in primordial and primary follicle oocytes to large gray structures in secondary follicle oocytes [49]. According to Isachenko et al. [86], these changes in appearance may be related to lipolysis, but they can also reflect a change in fatty acids composition as the oocyte develops [49]. This variation may be species-specific or related to factors such as the physiological status of the animal or its diet [87],[88]. Lipid droplets are also present in cattle [89] and sheep oocytes [90], though to a lesser extent.

The number of cytoplasmic vesicles increases in active oocytes in cattle [57] and buffaloes [45], occupying most of the oocyte cytoplasm. This increment might denote the stock of different biomolecules, like proteins, polysaccharide [63], or even lipids. In pigs, some structures first classified as vesicles were in fact lipid droplets, as proved by a specific stain method [49]. In cats, vesicles are scarce at this stage and in humans they appear especially at the antral stage [25]. Lucci et al. [36] suggested that some secretory vesicles may contain material for the synthesis of zona pellucida. The zona pellucida is made of glycoproteins, which are detected in the cytoplasm of follicular cells [91].

**Figure 5:**
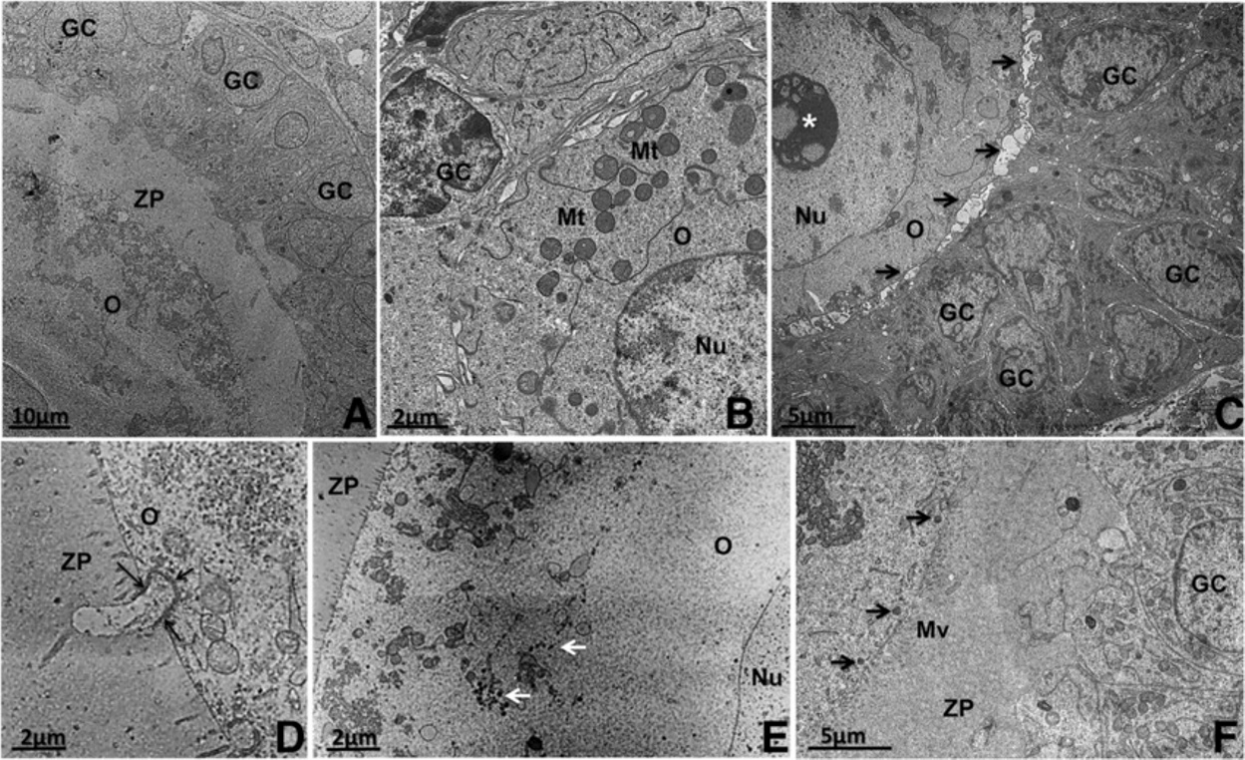
**Development of the zona pellucida** **(ZP)** **and cortical granules during follicular growth. A**: Cat primary follicle with a completely formed ZP. **B**: Pig primary follicle without ZP. **C**: Bitch small secondary follicle in which patches of ZP material start to be deposited around the oocyte (arrows). **D**: Detail of a granulosa cell projection through the ZP into the oocyte where gap junctions (arrows) can be seen in a pig secondary follicle. **E**: Cow large secondary follicle with cortical granules organized in clusters. Observe the organelle-free zone around the oocyte nucleus. **F**: Cat secondary follicle with cortical granules (arrows) aligned close to the oocyte plasma membrane. Note the microvilli on the oocyte plasma membrane protruding into the ZP. O: oocyte, Nu: nucleus, GC: granulosa cells, ZP: zona pellucida, *: nucleolus, Mt: mitochondria, Mv: Microvilli.

Zona pellucida is usually completely formed around the oocyte in secondary follicles, although in some species it has already developed at the primary follicle stage (Figure 5A). However, in species such as goats [47], buffaloes [45], yaks [52], pigs [49] and dogs [41] the zona pellucida is not yet visible in primary follicles (Figure 5B), or even in secondary follicles, in which only patches of zona pellucida material can be observed (Figure 5C). The formation of the zona pellucida is related to the appearance of short erect microvilli in the oocyte plasma membrane. Also, projections from granulosa cells are seen encroaching into the zona pellucida and protruding towards the oocyte, where gap junctions (Figure 5D) are found between oocyte and granulosa cell membranes [44],[57]. Gap junctions are responsible for intercommunication between oocytes and granulosa cells during the development of female gametes [92]. Evidence indicates that somatic cell-oocyte interactions via gap junctions are essential for oocyte growth and metabolism. So at this stage of follicle development coated pits are found in fairly small amounts [57].

Cortical granules are seen for the first time in secondary follicles. They are small organelles like vesicles containing enzymes that undergo exocytosis upon fertilization. At this time, cortical granules are aligned near the oocyte plasmatic membrane and the release of their contents aims to harden the zona pellucida to prevent polyspermy (for details see [93]). In secondary follicle oocytes, cortical granules usually appear in clusters (Figure 5E), either distributed all over the ooplasm or confined to the deep cortical area near the Golgi complex [57]. Exceptionally in the domestic cat these granules appear already aligned at the cortical region of the oocyte (Figure 5F) at the secondary follicle stage [39]. This feature, together with the early organization of organelles in clusters, suggests that in domestic cats the process of oocyte maturation occurs earlier than in other species [39], which may be related to their peculiarity of being a copulation-induced ovulation species. In non-domestic cats, the peripheral region of the ooplasm presents immature to mature cortical granules [59], and in cows small clusters of cortical granules were initially observed in large secondary follicle oocytes [44].

In general, the morphology of granulosa cells in secondary follicles resembles those in primary follicles. There are many electron-lucent vesicles in their cytoplasm in buffaloes and goats [45],[47]. Lucci et al. [47] suggest that granulosa cells are engaged in steroidogenesis, based on the great number of smooth endoplasmic reticulum and mitochondria present in their cytoplasm. Wolgemuth et al. [91] suggest that they are also involved in the synthesis of zona pellucida, because glycoproteins were identified in their cytoplasm.

**Figure 6:**
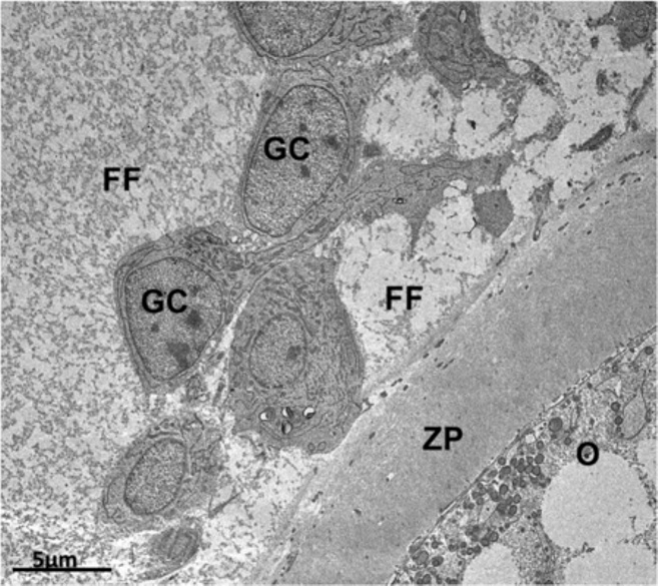
**Early antral follicle from pig showing spaces between adjacent granulosa cells filled with follicular fluid.** O: oocyte, ZP: zona pellucida, GC: granulosa cells, FF: folicular fluid.

The beginning of theca formation can be recognized by presence of elongated cells attached to the basement membrane, but the theca interna layer is still poorly defined in small secondary follicles [50]. On large secondary follicles, a clear theca interna layer is formed [50]. At this stage, spaces between adjacent granulosa cells filled with follicular fluid are also observed [47]. Progressive accumulation of fluid causes distension of these cavities and the initial formation of the antrum, leading the follicles to the antral stage [73] (Figure 6). The transition from preantral to early antral follicle is a critical stage of follicular development in terms of follicle destiny (growth versus atresia). During this period, the interaction between oocyte and somatic cells (granulosa and theca) is especially important, and many growth factors are involved (for a review see [94]).

### Antral formation and oocyte maturation

Antral formation occurs later in pig follicles (at 400 μm in diameter) [95] than in cattle (120–160 μm - [96]) and sheep follicles (220 μm - [97]; 300 μm - [98]). The differences in the timing of antrum formation may be important in the overall course of folliculogenesis, since there is a substantial increase in the growth rate of follicles after antrum formation. The fluid-filled antrum separates the cumulus *oophorus* cells surrounding the oocyte from the granulosa cells lining the wall of the follicle (for review, see [99]).

Mural granulosa cells of antral follicles are rich in Golgi complex, rough and smooth endoplasmic reticulum and small vesicles, as well as round and elongated mitochondria and lipid droplets [80]. Mural and cumulus granulosa cells of antral follicles are similar in ultrastructural organization, however they are different from preantral granulosa cells, having more smooth endoplasmic reticulum and lipid droplets, which suggest that they present different metabolic functions [80], developing mechanisms for producing steroid [100]. The granulosa membrane is separated from theca cells by collagen microfibrils. Cytoplasmic contact between theca and granulosa cells was never seen. Theca interna cells have an elongated nucleus. The number of mitochondria, rough endoplasmic reticulum and free ribosomes vary among individual theca cells, and seems to increase as they became more differentiated. Golgi complexes associated with many small vesicles are always present [101],[102]. Capillaries are often seen in the theca interna, specially concentrated close to the basal lamina [101],[102]. A larger number of capillaries of different sizes are frequently observed in the theca externa [102].

**Figure 7:**
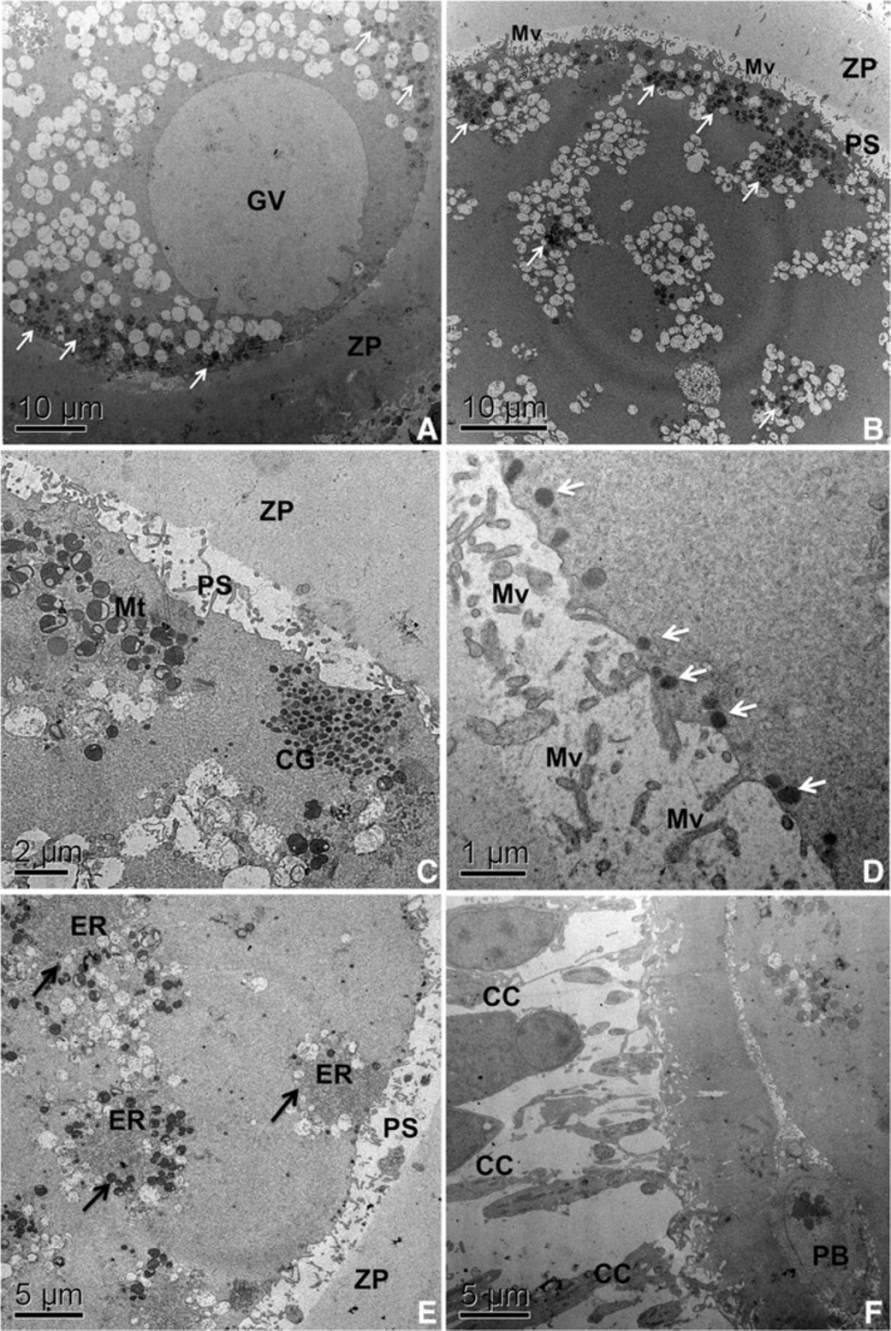
**Ultrastructural events during oocyte maturation in bovine. A**: Oocyte from tertiary follicle with intact germinal vesicle (GV), showing mitochondria (arrows) in peripheral position. Note the great amount at vesicles throughout the ooplasm. **B**: Oocyte after 12 hours of in vitro maturation (IVM) presenting mitochondria clustered (arrows) mostly at cortical areas. Microvilli loosen from the zona pellucida. Observe the general organization of organelles in small groups. **C**: Oocyte after 12 hours of IVM. Cortical granules clusters are located at periphery of the ooplasma, close to the plasma membrane. Note a group of hooded/pleomorphic mitochondria. **D**: Oocyte after 18 hours of IVM showing cortical granules (arrows) aligned to the plasma membrane. **E**: oocyte after 18 hours of IVM. Observe the peculiar arrangement of organelles, with endoplasmic reticulum in close association with mitochondria and vesicles (arrows). **F**: mature oocyte after 24 hours of IVM that have extruded the first polar body (PB). Note the expanded cumulus cells. GV: germinal vesicle, PS: perivitelline space, ZP: zona pelucida, Mv: microvilli, Mt: mitochondria, CG: cortical granules, ER: endoplasmic reticulum, CC: cumulus cells, PB: polar body.

In general, in tertiary follicles, all the oocytes are completely surrounded by the zona pellucida, which is crossed by projections of the granulosa cells that form indentations in the oolemma [57]. At this time, the organelles have achieved a more even distribution throughout the ooplasm, and elongated mitochondria, lipid droplets and vesicles increase in numbers [66] (Figure 7A). That is only reasonable, since oocytes that grow to a bigger size may require larger amounts of the machinery needed to move and store cytoplasmic constituents [56].

Large amounts of lipids in oocytes are observed isolated or organized in groups in mouse [103]. In buffaloes these lipid droplets have been confirmed by the addition of the component thiol in the culture medium of *in vitro* maturation [104]. In oocytes derived from buffalo follicles (6 mm in diameter) organelles are located in the perinuclear region, mitochondria in the cortical area and lipid droplets in the medullary area [34]. The authors suggested that this organization indicates a high metabolic rate of these oocytes, which tends to increase with its development and growth.

Several ultrastructural changes can be observed in cytoplasmic organelles during oocyte maturation. Mitochondria move from a peripheral position (Figure 7A) before the luteinizing hormone (LH) surge to a scatter distribution throughout the cytoplasm (not shown) and have a clustered cortical formation in the final stages of nuclear maturation (Figure 7B), and a dispersed distribution after the extrusion of the polar body [77]. At that time oocyte microvilli loosen from the zona pellucida (Figure 7B). Upon reaching metaphase II the mitochondria and lipid droplets occupy a central position in the cell [66].

Cortical granules that were arranged in clusters in the deep cortex of secondary follicle oocytes [53] progressively migrate towards the subplasmalemmal areas in antral follicle oocytes (Figure 7C) [105],[106]. Cortical granules are derived from the Golgi complex and continuously produced until ovulation [107], and their migration to the periphery of the oocyte is an important step in oocyte cytoplasmic maturation [108]. At the end of the maturation period, when these oocytes reach metaphase II, cortical granules are aligned to the inner surface of the oocyte plasma membrane (Figure 7D) [109],[110], ready to released their contents as soon as the oocyte is fertilized to prevent polyspermy [93].

Furthermore, the cytoplasm of the oocyte from tertiary follicles is characterized by the presence of hooded and pleomorphic mitochondria, and well developed Golgi cisternae, mainly in the periphery of the ooplasm [111]. The dynamics of the Golgi membranes during maturation and fertilization in mammals requires more study. Associations between endoplasmic reticulum, mitochondria and lipid droplets become common (Figures 4C and 7E) [66],[77]. This organelles association is both related to lipid metabolism and ER-mitochondria calcium signaling [61]. It allows efficient transmission of signals from cytosolic calcium to the mitochondria, enabling activation of the mitochondrial metabolism and an increase in ATP supply for the calcium pump in the endoplasmic reticulum [112],[113]. It is likely that in oocytes at this stage of development, this structure is involved in the regulation of sperm-triggered Ca^2+^ oscillation [112]. The membranes of the endoplasmic reticulum are physiologically active and interact with the cytoskeleton [114]. The endoplasmic reticulum reorganization in oocyte maturation is a complex multistep process involving distinct microtubule and microfilament-dependent phases [115].

The mature oocyte is finally ovulated usually at the metaphase II stage, having extruded the first polar body (Figure 7F). Of course, all those morphological changes happen concomitantly with biochemical and molecular modifications (for details see [114],[116]), which lead the oocytes to nuclear and cytoplasmic maturation and guarantee their competence to be fertilized.

## Conclusions

In recent decades, the understanding of reproductive physiology in mammals has shown great advances, especially in respect to preantral follicles. Many morphological and ultrastructural aspects of oocytes have been identified, allowing a better understanding of their physiology.

The knowledge of ultrastructural changes oocytes must undergo to develop normally and become competent may aid in the development of female gamete manipulation techniques, such as *in vitro* maturation of oocytes and *in vitro* culture of preantral follicles. Nowadays, these techniques work better in some species than others, and any new information or elucidation of species-specific differences may be important for further improvements, helping in the understanding of damage and in surpassing limitations.

## Authors' contributions

FP drafted the manuscript and participated in the morphological analysis. RCS and JLJdePR carried out many of the electron microscopy processing, analysis and image acquisition. CML conceived, designed and coordinated the study and helped to draft the manuscript. All authors read and approved the final manuscript.

## Authors’ original submitted files for images

Below are the links to the authors’ original submitted files for images. Authors’ original file for figure 1 Authors’ original file for figure 2 Authors’ original file for figure 3 Authors’ original file for figure 4 Authors’ original file for figure 5 Authors’ original file for figure 6 Authors’ original file for figure 7

## References

[1] Koering MJ (1969). Cyclic changes in ovarian morphology during the menstrual cycle in Macaca mulatta. Am J Anat.

[2] VanWezel IL, Rodgers RJ (1996). Morphological characterization of bovine primordial follicles and their environment in vivo. Biol Reprod.

[3] Hulshof SCJ, Figueiredo JR, Beckers JF, Bevers MM, Vandenhurk R (1994). Isolation and Characterization of Preantral Follicles from Fetal Bovine Ovaries. Vet Quart.

[4] Hsueh AJW, Adashi EY, Jones PBC, Welsh TH (1984). Hormonal-Regulation of the Differentiation of Cultured Ovarian Granulosa-Cells. Endocr Rev.

[5] Richards JS (1994). Hormonal control of gene expression in the ovary. Endocr Rev.

[6] Chun SY, Hsueh AJW (1998). Paracrine mechanisms of ovarian follicle apoptosis. J Reprod Immunol.

[7] Roy SK, Greenwald GS (1989). Hormonal requirements for the growth and differentiation of hamster preantral follicles in long-term culture. J Reprod Fertil.

[8] Nayudu PL, Osborn SM (1992). Factors influencing the rate of preantral and antral growth of mouse ovarian follicles in vitro. J Reprod Fertil.

[9] Cain L, Chatterjee S, Collins TJ (1995). In-Vitro Folliculogenesis of Rat Preantral Follicles. Endocrinology.

[10] Picton HM (2001). Activation of follicle development: The primordial follicle. Theriogenology.

[11] Wassarman PM, Josefowicz WJ (1978). Oocyte Development in Mouse - Ultrastructural Comparison of Oocytes Isolated at Various Stages of Growth and Meiotic Competence. J Morphol.

[12] Aerts JMJ, Bols PEJ (2010). Ovarian Follicular Dynamics. A review with Emphasis on the Bovine Species. Part II: Antral Development, Exogenous Influence and Future Prospects. Reprod Domest Anim.

[13] Rüsse ISF (1991). Gametogenese & Harn- und Geschlechtsorgane.

[14] Peters H (1976). The development and maturation of the ovary. Ann Biol Anim Bioch Biophys.

[15] Gosden RGBM (1995). Cellular and molecular aspects of oocyte development.

[16] Gosden B (1978). Oogonia and oocytes in mammals.

[17] Johnson J, Canning J, Kaneko T, Pru JK, Tilly JL (2004). Germline stem cells and follicular renewal in the postnatal mammalian ovary (vol 428, pg 145, 2004). Nature.

[18] Notarianni E (2011). Reinterpretation of evidence advanced for neo-oogenesis in mammals, in terms of a finite oocyte reserve. J Ovarian Res.

[19] White YAR, Woods DC, Takai Y, Ishihara O, Seki H, Tilly JL (2012). Oocyte formation by mitotically active germ cells purified from ovaries of reproductive-age women. Nat Med.

[20] Erickson BH (1966). Development And Radio-Response Of The Prenatal Bovine Ovary. J Reprod Fertil.

[21] Black JL, Erickson BH (1968). Oogenesis and ovarian development in the prenatal pig. Anat Rec.

[22] El-Ghannam F, El-Naggar MA (1974). The prenatal development of the buffalo ovary. J Reprod Fertil.

[23] Beaumont HM, Mandl AM (1962). A Quantitative and Cytological Study of Oogonia and Oocytes in the Foetal and Neonatal Rat. Proc Royal Soc London Series B, Biol Sci.

[24] Baker TG (1963). A Quantitative and Cytological Study of Germ Cells in Human Ovaries. Proc Royal Soc London Series B, Biol Sci.

[25] Sathananthan AH, Selvaraj K, Girijashankar ML, Ganesh V, Selvaraj P, Trounson AO (2006). From oogonia to mature oocytes: inactivation of the maternal centrosome in humans. Microsc Res Tech.

[26] Saumande J (1991). Folliculogenesis in Ruminants. Recl Med Vet.

[27] Saumande J (1981). Oogenesis and Folliculogenesis. Recl Med Vet.

[28] Peters H, McNatty KP (1980). The Ovary: A Correlation of Structure and Function in Mammals. Morphology of the ovary.

[29] Peters H (1969). The development of the mouse ovary from birth to maturity. Acta Endocrinol.

[30] Merchant-Larios H, Chimal-Monroy J (1989). The ontogeny of primordial follicles in the mouse ovary. Prog Clin Biol Res.

[31] Vaskivuo T (2002). Regulation of apoptosis in the female reproductive system.

[32] Lucci CM, Rumpf R, Figueiredo JR, Bao SN (2002). Zebu (Bos indicus) ovarian preantral follicles: morphological characterization and development of an efficient isolation method. Theriogenology.

[33] Silva-Santos KC, Santos GM, Siloto LS, Hertel MF, Andrade ER, Rubin MI, Sturion L, Melo-Sterza FA, Seneda MM (2011). Estimate of the population of preantral follicles in the ovaries of Bos taurus indicus and Bos taurus taurus cattle. Theriogenology.

[34] Mondadori RG, Santin TR, Fidelis AA, Porfirio EP, Bao SN (2010). Buffalo (Bubalus bubalis) pre-antral follicle population and ultrastructural characterization of antral follicle oocyte. Reprod Domest Anim.

[35] Land RB (1970). Number Of Oocytes Present At Birth In The Ovaries Of Pure And Finnish Landrace Cross Blackface And Welsh Sheep. J Reprod Fertil.

[36] Lucci CM, Amorim CA, Rodrigues APR, Figueiredo JR, Báo SN, Silva JRV, Gonçalves PBD (1999). Study of preantral follicle population in situ and after mechanical isolation from caprine ovaries at different reproductive stages. Anim Reprod Sci.

[37] Gougeon A, Ecochard R, Thalabard JC (1994). Age-related changes of the population of human ovarian follicles: increase in the disappearance rate of non-growing and early-growing follicles in aging women. Biol Reprod.

[38] Domingues SFS, Diniz LV, Furtado SHC, Ohashi OM, Rondina D, Silva LDM (2004). Histological study of capuchin monkey (Cebus apella) ovarian follicles. Acta Amazonica.

[39] Carrijo Jr OA, Marinho AP, Campos AA, Amorim CA, Bao SN, Lucci CM (2010). Morphometry, estimation and ultrastructure of ovarian preantral follicle population in queens. Cells Tissues Organs.

[40] Erickson BH (1967). Radioresponse of Pre-puberal Porcine Ovary. Int J Radiat Biol Relat Stud Phys Chem Med.

[41] Jivago JLPR: **Estudo da populaçäo e criopreservaçäo de folículos ovarianos pré-antrais de cadelas**. *Master of Science Thesis.* University of Brasilia: Graduation Program in Animal Biology; 2012.

[42] Rodgers RJ, Irving-Rodgers HF (2010). Morphological classification of bovine ovarian follicles. Reproduction.

[43] Ross M, Romell LJ, Kaye GI (1995). Histology: A Text and Atlas.

[44] Kacinskis MA, Lucci CM, Luque MC, Bao SN (2005). Morphometric and ultrastructural characterization of Bos indicus preantral follicles. Anim Reprod Sci.

[45] Mondadori RG, Luque MC, Santin TR, Bao SN (2007). Ultrastructural and morphometric characterization of buffalo (Bubalus bubalis) ovarian preantral follicles. Anim Reprod Sci.

[46] Lundy T, Smith P, O'Connell A, Hudson NL, McNatty KP (1999). Populations of granulosa cells in small follicles of the sheep ovary. J Reprod Fertil.

[47] Lucci CM, Silva RV, Carvalho CA, Figueiredo R, Báo N (2001). Light microscopical and ultrastructural characterization of goat preantral follicles. Small Ruminant Res.

[48] Gougeon A, Chainy GBN (1987). Morphometric Studies of Small Follicles in Ovaries of Women at Different Ages. J Reprod Fertil.

[49] Silva RC, Bao SN, Jivago JL, Lucci CM (2011). Ultrastructural characterization of porcine oocytes and adjacent follicular cells during follicle development: lipid component evolution. Theriogenology.

[50] Braw-Tal R, Yossefi S (1997). Studies in vivo and in vitro on the initiation of follicle growth in the bovine ovary. J Reprod Fertil.

[51] Yang YJ, Zhang YJ, Li Y (2009). Ultrastructure of human oocytes of different maturity stages and the alteration during in vitro maturation. Fertil Steril.

[52] Yu SJ, Yong YH, Cui Y (2010). Oocyte morphology from primordial to early tertiary follicles of yak. Reprod Domest Anim.

[53] Fair T, Hulshof SCJ, Hyttel P, Greve T, Boland M (1997). Nucleus ultrastructure and transcriptional activity of bovine oocytes in preantral and early antral follicles. Mol Reprod Dev.

[54] de Bruin JP, Dorland M, Spek ER, Posthuma G, van Haaften M, Looman CWN, te Velde ER (2002). Ultrastructure of the resting ovarian follicle pool in healthy young women. Biol Reprod.

[55] Hertig AT, Adams EC (1967). Studies on the human oocyte and its follicle. I. Ultrastructural and histochemical observations on the primordial follicle stage. J Cell Biol.

[56] Pepling ME, Wilhelm JE, O'Hara AL, Gephardt GW, Spradling AC (2007). Mouse oocytes within germ cell cysts and primordial follicles contain a Balbiani body. Proc Natl Acad Sci U S A.

[57] Fair T, Hulshof SCJ, Hyttel P, Greve T, Boland M (1997). Oocyte ultrastructure in bovine primordial to early tertiary follicles. Anat Embryol.

[58] Greenwald GS, Moor RM (1989). Isolation and preliminary characterization of pig primordial follicles. J Reprod Fertil.

[59] Jewgenow K, Stolte M (1996). Isolation of preantral follicles from nondomestic cats—viability and ultrastructural investigations. Anim Reprod Sci.

[60] Brown DA (2001). Lipid droplets: Proteins floating on a pool of fat. Curr Biol.

[61] Raturi A, Simmen T (1833). Where the endoplasmic reticulum and the mitochondrion tie the knot: the mitochondria-associated membrane (MAM). Biochim Biophys Acta.

[62] Reader KL: **A Quantitative Ultrastructural Study of Oocytes During the Early Stages of Ovarian Follicular Development in Booroola and Wild-Type Sheep.** *Master of Science thesis.* Victoria University of Wellington; 2007

[63] Guraya SS, Monga S, Kaur P, Sangha GK (1998). Comparative morphological and histochemical studies of ovarian follicles in the goat and sheep. Indian J Anim Sci.

[64] Fawcett DW (1966). An atlas of fine structure. The cell. Its organelles and inclusions.

[65] Fair T (1995). Oocyte growth in cattle: Ultrastructure, transcription and developmental competence.

[66] Hyttel P, Fair T, Callesen H, Greve T (1997). Oocyte growth, capacitation and final maturation in cattle. Theriogenology.

[67] Hirshfield AN (1991). Development of Follicles in the Mammalian Ovary. Int Rev Cytol.

[68] Hage AJ, Groen-Klevant AC, Welschen R (1978). Follicle growth in the immature rat ovary. Acta Endocrinol.

[69] Odor DL (1960). Electron microscopic studies on ovarian oocytes and unfertilized tubal ova in the rat. J Biophys Biochem Cytol.

[70] Oakberg EF (1979). Follicular growth and atresia in the mouse. In Vitro.

[71] Adams EC, Hertig AT (1964). Studies on Guinea Pig Oocytes. I. Electron Microscopic Observations on the Development of Cytoplasmic Organelles in Oocytes of Primordial and Primary Follicles. J Cell Biol.

[72] Nicosia SV, Evangelista I, Batta SK (1975). Rabbit Ovarian Follicles. I. Isolation Technique and Characterization at Different Stages of Development. Biol Reprod.

[73] Zamboni L (1974). Fine Morphology of Follicle Wall and Follicle Cell Oocyte Association. Biol Reprod.

[74] Himelstein-Braw R, Byskov AG, Peters H, Faber M (1976). Follicular atresia in the infant human ovary. J Reprod Fertil.

[75] Newton H, Aubard Y, Rutherford A, Sharma V, Gosden R (1996). Low temperature storage and grafting of human ovarian tissue. Hum Reprod.

[76] Matzuk MM, Burns KH, Viveiros MM, Eppig JJ (2002). Intercellular communication in the mammalian ovary: oocytes carry the conversation. Science.

[77] Kruip TAM, Cran DG, van Beneden TH, Dieleman SJ (1983). Structural changes in bovine oocytes during final maturation in vivo. Gamete Res.

[78] Hyttel P, Xu KP, Smith S, Greve T (1986). Ultrastructure of Invitro Oocyte Maturation in Cattle. J Reprod Fertil.

[79] Hay M, Cran DG, Moor RM (1976). Structural changes occurring during atresia in sheep ovarian follicles. Cell Tissue Res.

[80] Sharma RK, Sawhney AK (1999). Fine morphology of membrana granulosa in caprine ovary. Indian J Anim Sci.

[81] Westergaard CG, Byskov AG, Andersen CY (2007). Morphometric characteristics of the primordial to primary follicle transition in the human ovary in relation to age. Hum Reprod.

[82] Assey RJ, Hyttel P, Roche JF, Boland MP (1994). Infrequent Structures in Cattle Oocytes. Anat Embryol.

[83] Cran DG, Moor RM, Hay MF (1980). Fine-Structure of the Sheep Oocyte during Antral Follicle Development. J Reprod Fertil.

[84] Russe I (1975). Symposium on scanning electron microscopy of fertility and infertility, Eighth World Congress of Fertility and Sterility, Buenos Aires, Argentina, November 1974. Unfertilized sheep eggs. J Reprod Med.

[85] Fleming WN, Saacke RG (1972). Fine structure of the bovine oocyte from the mature graafian follicle. J Reprod Fertil.

[86] Isachenko V, Isachenko E, Michelmann HW, Alabart JL, Vazquez I, Bezugly N, Nawroth F (2001). Lipolysis and ultrastructural changes of intracellular lipid vesicles after cooling of bovine and porcine GV-oocytes. Anat Histol Embryol.

[87] Adamiak SJ, Mackie K, Watt RG, Webb R, Sinclair KD (2005). Impact of nutrition on oocyte quality: cumulative effects of body composition and diet leading to hyperinsulinemia in cattle. Biol Reprod.

[88] Leroy JLMR, Vanholder T, Delanghe JR, Opsomer G, Van Soom A, Bols PEJ, de Kruif A (2004). Metabolite and ionic composition of follicular fluid from different-sized follicles and their relationship to serum concentrations in dairy cows. Anim Reprod Sci.

[89] Basso AC, Esper CR (2002). Isolation and ultrastructural characterization of preantral follicles in the Nelore breed cows (Bos Taurus indicus). Braz J Vet Res Anim Sci.

[90] Andrade ER, Maddox-Hyttel P, Landim-Alvarenga Fda C, Viana Silva JR, Alfieri AA, Seneda MM, Figueiredo JR, Toniolli R (2010). Ultrastructure of Sheep Primordial Follicles Cultured in the Presence of Indol Acetic Acid, EGF, and FSH. Vet Med Int.

[91] Wolgemuth DJ, Celenza J, Bundman DS, Dunbar BS (1984). Formation of the rabbit zona pellucida and its relationship to ovarian follicular development. Dev Biol.

[92] Grazul-Bilska AT, Reynolds LP, Redmer DA (1997). Gap junctions in the ovaries. Biol Reprod.

[93] Liu M (2011). The biology and dynamics of mammalian cortical granules. Reprod Biol Endocrinol: RB&E.

[94] Orisaka M, Tajima K, Tsang BK, Kotsuji F (2009). Oocyte-granulosa-theca cell interactions during preantral follicular development. J Ovarian Res.

[95] Morbeck DE, Esbenshade KL, Flowers WL, Britt JH (1992). Kinetics of Follicle Growth in the Prepubertal Gilt. Biol Reprod.

[96] Lussier JG, Matton P, Dufour JJ (1987). Growth rates of follicles in the ovary of the cow. J Reprod Fertil.

[97] Cahill LP, Mauleon P (1980). Influences of Season, Cycle and Breed on Follicular-Growth Rates in Sheep. J Reprod Fertil.

[98] Turnbull KE, Braden AW, Mattner PE (1977). The pattern of follicular growth and atresia in the ovine ovary. Aust J Biol Sci.

[99] Motta PM, Nottola SA, Familiari G, Makabe S, Stallone T, Macchiarelli G (2003). Morphodynamics of the follicular-luteal complex during early ovarian development and reproductive life. Int Rev Cytol - a Surv Cell Biology.

[100] Ax RL, Bushmeyer SM, Boehm SK, Bellin ME (1984). Binding of the glycosaminoglycan [3H]heparin to bovine granulosa cells varies with size and estrogen content of ovarian follicles. Endocr Res.

[101] O'Shea JD, Cran DG, Hay MF, Moor RM (1978). Ultrastructure of the theca interna of ovarian follicles in sheep. Cell Tissue Res.

[102] Sharma RK, Sawhney AK, Vats R (1996). Ultrastructure of thecal components in caprine ovary. Small Ruminant Res.

[103] Dvorak M (1989). Ultrastructure and quantitative analysis of mouse and human oocytes. Prog Clin Biol Res.

[104] Gasparrini B, Boccia L, Marchandise J, Di Palo R, George F, Donnay I, Zicarelli L (2006). Enrichment of in vitro maturation medium for buffalo (Bubalus bubalis) oocytes with thiol compounds: effects of cystine on glutathione synthesis and embryo development. Theriogenology.

[105] Baca M, Zamboni L (1967). The fine structure of human follicular oocytes. J Ultrastruct Res.

[106] Sathananthan AH, Trounson AO (1985). The human pronuclear ovum: Fine strcture of monospermic and polyspermic fertilization in vitro. Gamete Res.

[107] Gulyas BJ (1980). Cortical granules of mammalian eggs. Int Rev Cytol.

[108] Damiani P, Fissore RA, Cibelli JB, Long CR, Balise JJ, Robl JM, Duby RT (1996). Evaluation of developmental competence, nuclear and ooplasmic maturation of calf oocytes. Mol Reprod Dev.

[109] Conner S, Leaf D, Wessel G (1997). Members of the SNARE hypothesis are associated with cortical granule exocytosis in the sea urchin egg. Mol Reprod Dev.

[110] Thibault C, Szollosi D, Gerard M (1987). Mammalian oocyte maturation. Reprod Nutr Dev.

[111] Assey RJ, Hyttel P, Kanuya N (1994). Oocyte Structure in Dominant and Subordinate Follicles in Zebu Cattle (Bos-Indicus). Anat Embryol.

[112] Dumollard R, Duchen M, Carroll J (2007). The role of mitochondrial function in the oocyte and embryo. Curr Top Dev Biol.

[113] Rizzuto R, Bernardi P, Pozzan T (2000). Mitochondria as all-round players of the calcium game. J Physiol.

[114] Ferreira EM, Vireque AA, Adona PR, Meirelles FV, Ferriani RA, Navarro PA (2009). Cytoplasmic maturation of bovine oocytes: structural and biochemical modifications and acquisition of developmental competence. Theriogenology.

[115] Mehlmann LM, Terasaki M, Jaffe LA, Kline D (1995). Reorganization of the Endoplasmic Reticulum during Meiotic Maturation of the Mouse Oocyte. Dev Biol.

[116] Mao L, Lou H, Lou Y, Wang N, Jin F (2014). Behaviour of cytoplasmic organelles and cytoskeleton during oocyte maturation. Reprod Biomed Online.

