# Supplementary figures and images for: Ultrastructural changes in oocytes during folliculogenesis in domestic mammals
Source: J Ovarian Res. 2014 Oct 30;7:102. doi: 10.1186/s13048-014-0102-6 (PMC4224757; doi:10.1186/s13048-014-0102-6)

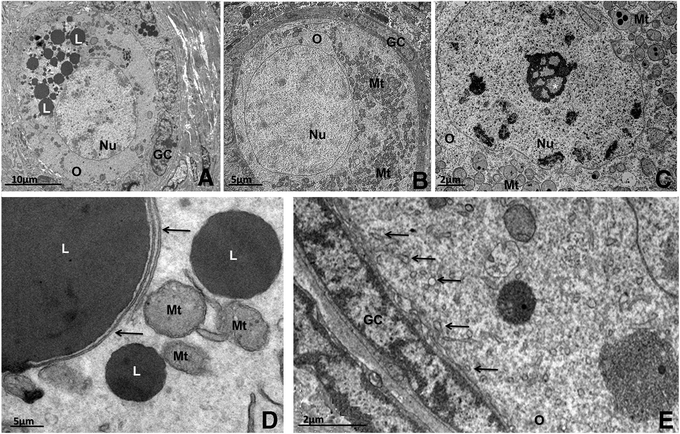

Supplement: Supplementary file 1 — Authors’ original file for figure 1 [file 13048_2014_102_MOESM1_ESM.gif]

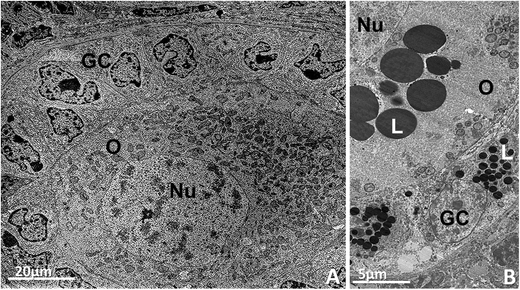

Supplement: Supplementary file 2 — Authors’ original file for figure 2 [file 13048_2014_102_MOESM2_ESM.gif]

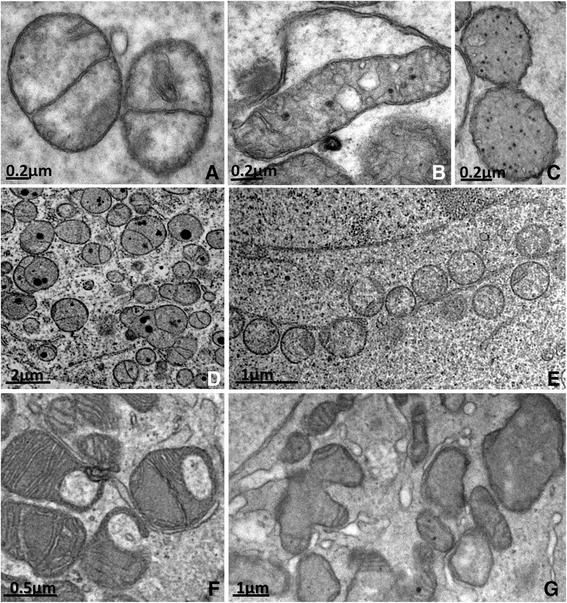

Supplement: Supplementary file 3 — Authors’ original file for figure 3 [file 13048_2014_102_MOESM3_ESM.gif]

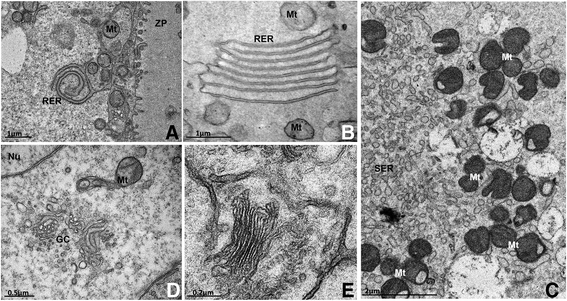

Supplement: Supplementary file 4 — Authors’ original file for figure 4 [file 13048_2014_102_MOESM4_ESM.gif]

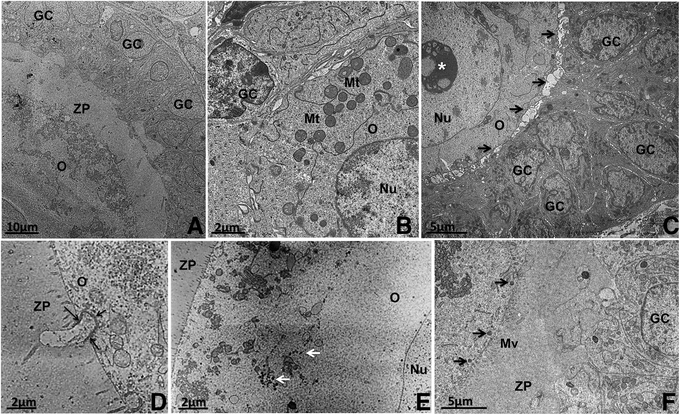

Supplement: Supplementary file 5 — Authors’ original file for figure 5 [file 13048_2014_102_MOESM5_ESM.gif]

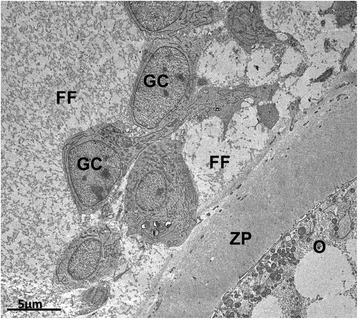

Supplement: Supplementary file 6 — Authors’ original file for figure 6 [file 13048_2014_102_MOESM6_ESM.gif]

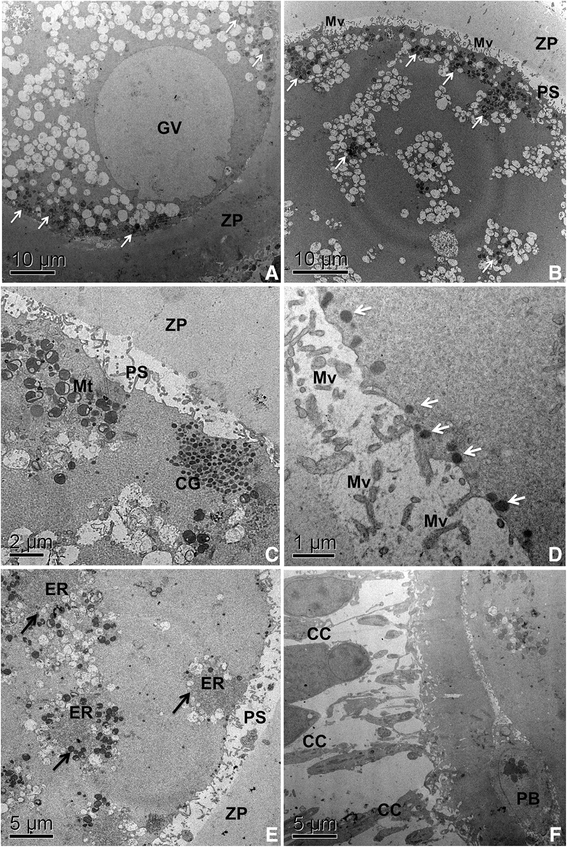

Supplement: Supplementary file 7 — Authors’ original file for figure 7 [file 13048_2014_102_MOESM7_ESM.gif]
